# History of a Case of the Successful Formation of an Artificial Anus in an Adult with an Account of an Analogous Operation in Two Cases

**Published:** 1821-01

**Authors:** Daniel Pring, G. Freer

**Affiliations:** Member of the College of Surgeons in London; Birmingham.


### Original Communications, Select Observations, etc.

### *History of a Case of the successful Formation of an Artificial Anus in an Adult*, by DANIEL PRING, Member of the College of Surgeons in London: *with an Account of an analogous Operation in two Cases*, by G. FREER, Esq. of Birmingham.

THE case which forms a principal subject of the following memoir, derives the little value and interest it might possess chiefly from the two circumstances, that there are but few examples of a similar practice in surgery, and that the one here related exhibits a more successful result than general experience would incline us to anticipate.

Mrs. White, who is a widow, but has never borne children, is sixty-four years of age; she is rather of a full habit, with a florid complexion; her health has generally been pretty good, although she has not, at former periods of her life, been exempt from severe indispositions, principally affecting her head. About the middle of the summer of 1819, she was first troubled with acute pains in the abdomen, the general tenderness of which was much increased by pressure; more particularly so on the left side, a little below the ribs. Her stools often contained blood, with a large proportion of mucus or lymph, and were otherwise commonly of a very bad quality. Her disease was considered as dysenteric, and was treated principally by mercury, ipecacuanha, opium, and saline aperients. In the course of a fortnight from the commencement of this plan her gums became affected by the mercury, although the quantity of the blue-pill which she had taken did not, upon an average, exceed from five to ten grains a-day; her teeth were loose, and an ulcer formed on one cheek. The mercury was discontinued for a week; and, as her symptoms had before improved under it, the same treatment was then resumed for another week, when, the tongue and cheek becoming sore, it was again discontinued. The mouth speedily recovered from the mercurial affection, and she left Bath for her residence in Gloucestershire, apparently in good health, at least with very little remains of her former symptoms. I was informed that, about the Christmas following, she had suffered an attack of inflammation of the bowels, with obstinate constipation. Her bowels had for some time previously been occasionally painful, and the discharges from them irregular: she had none but fluid stools, and, when not suffering a spontaneous diarrhœa, was under the necessity of taking purgative medicines; to the neglect of which was imputed the attack of inflammation which occurred about Christmas. Recovering from this attack, she relapsed into her former state of chronic disease, of a dysenteric character.

Mrs. White returned to Bath in February, 1820, and a medical treatment, very little differing from that before described, was again instituted, apparently with some benefit. She first mentioned to me, in the beginning of March, that she felt an obstruction in the lower bowels: every thing, she said, appeared to stop at one place; and, when the contents of the bowels passed beyond this point, she was relieved from the distress of pain and distention which she before felt. She described the seat of the obstruction as being very low. I examined the rectum with my finger, and, as high as it would reach, I could perceive nothing like disease. I then attempted to introduce a rectum-bougie, which passed freely about four inches up the gut, and then met with some impediment. The bougie was introduced every second day, and, on the third or fourth introduction, it passed freely beyond that which I had supposed was a stricture; but, whether this contraction had previously existed, or was excited by the irritation of the bougie, I do not feel competent to determine. In subsequent examinations, a middle-sized rectum-bougie passed readily about seven inches; its further progress was completely impeded. It was attempted to introduce a large-sized urethra-bougie: this also stopped at the same place, and bent with the pressure employed to introduce it further. After reiterated trials with bougies of various sizes, the result was so little satisfactory, that no advantage could be reasonably expected from persevering in this plan of treatment: it was therefore abandoned. For some weeks afterwards, the symptoms underwent very little variety. As long as the bowels were kept open by the regular use of castor oil, Epsom salts, &c. the distress of the symptoms was mitigated: but, these means failing, as they sometimes did, a constipation ensued, which was with difficulty overcome by the most powerful purgatives, aided by clysters with soap, aloes, &c.

With great difficulty some small purging stools had been procured almost every day, up to the 25th of June, when the obstruction, which had long been increasing, appeared to have become complete. All the medical resources of art were afterwards exhausted in fruitless attempts to procure evacuations. Salts, senna, aloes, colocynth, jalap, scammony, gamboge, elaterium, calomel, were given in their largest doses, and variously combined: castor oil was also given in doses of three ounces; and, as vomiting was by no means frequent, these medicines were commonly retained. Injections of every sort, and by different means, were also administered: they were sometimes retained for about half an hour, to the amount of four or six ounces, and were then forcibly expelled. It was attempted to pass a flexible catheter beyond the obstruction, through which clysters might be thrown into the bowels above the seat of it: whether this attempt succeeded in its mechanical object, was not certain. The pulse generally, during the period of this obstruction, was about 90, seldom exceeding 100: the tongue was dry, but clean; there was no vomiting, unless this action was excited by the presence of medicines or food; there was considerable distention, and some tenderness, of the abdomen, but the degree of the latter was not very great. She was once bled, without any relief to her symptoms, rather as a measure of prevention than of necessity; and, when all other means had failed, some large doses of laudanum were given, without any reasonable expectation, on the supposed possibility of the existence of spasm.

It appeared quite clear, after the persevering trial of the above means for many days, that the obstruction, by whatever cause produced, was one which could not be overcome. A somewhat similar case, of a medical practitioner in this city, had a short time before terminated fatally; which case was reported in your Journal by Mr. White. Anticipating that the case of my patient might soon become one of equal extremity, I determined to give her a chance for her life, by making an artificial anus, if she was willing that her life should be prolonged upon such terms. I had heard vaguely, by report, that such an operation had been resorted to by Mr. Freer, of Birmingham, to whose example and instruction I have always much pleasure in acknowledging my obligation: but I was not, at that time, acquainted with the particulars of his operation, or with the exact nature of the disease for the relief of which it was instituted. I accordingly proposed this dernier resort to my patient, who did not violently object to it. I wished her, however, to have another opinion upon her case before she decided on this question; and was happy to avail myself of the well-known professional judgment and skill of Mr. George Norman. I met Mr. Norman in consultation on the 6th of July; and, after a very long and accurate investigation of the nature of the disease, so far as it could be judged of by its history, and by examination of the rectum, both with the finger and with bougies, Mr. Norman concurred with me in thinking that nothing was to be gained by a longer trial of medicines; and also that the case, if allowed a spontaneous course, must speedily have a fatal termination. Mr. Norman also agreed in thinking that an artificial anus offered the only resource for prolonging her life, but thought it eligible to wait twenty-four hours longer.

The following day, the 7th of July, twelve days from the commencement of the total retention of feces, I was accompanied by Mr. George Skinner, on whom a considerable share of the treatment subsequently devolved, and again met Mr. Norman. We found that the state of our patient was not at all improved, but rather that she was sinking under her protracted disease. The formation of an artificial anus was again proposed, and all its possible advantages and disadvantages fairly stated. The patient was desirous of living upon any terms, and agreed to submit to the operation. Accordingly, having placed her on a table, I made an incision on the left side of the abdomen, beginning about two inches above, and one inch on the inside, of the anterior superior spinous process of the ilium. This incision was extended obliquely downwards and inwards to within three-quarters of an inch of the edge of Poupart's ligament: the fascia covering the abdominal muscles was thus exposed to the extent of between three and four inches. An opening was then made through the external and internal oblique, and the transversalis, muscles; which opening was enlarged, with a bistoury conducted by my finger, to the extent of the external incision. The peritoneum being now laid bare, a small opening was made in this membrane, which was enlarged to the extent of between two and three inches. The patient being greatly agitated, and the diaphragm and abdominal muscles thrown into violent action, a considerable protrusion occurred at this time of the small intestines from the superior part of the wound: these were, however, readily replaced. The colon was thus freely exposed a little above its sigmoid flexure, at which place I made an incision into it of about an inch and a half in length: this incision was made on the left side of the gut, in order to avoid some small branches of the inferior mesenteric artery, which were distributed on the right. The contents of the bowels were immediately expelled with great force to a considerable distance: as the feces escaped, the gut collapsed, and began to subside from its place: a ligature was therefore passed through it at the lower part of the opening, the apposition of which to the external wound was then preserved until the bowels were copiously evacuated. The obstruction could not be felt by the finger, introduced through the wound: no attempt was therefore made to overcome it in this direction. The opening in the gut was attached to the external wound by four sutures, one in its superior and inferior margins, and one on each side; and the wound of the integument was closed by two sutures above, and one below, the opening of the intestine: it was then dressed with sticking-plaster above and below the opening into the gut, and this latter place was covered by a light compress; the patient was then put into bed.

Very little disturbance of the constitution succeeded immediately to the operation. There was no vomiting: the patient felt relieved from the pain of distention; passed liquid stools frequently; took gruel; and slept a little in the course of the night. The following day, the wound looked well: the pulse was between 100 and 110; the tongue dry; there was very little soreness of the abdomen. She took some aperient medicine, gruel, and beef-tea. The wound, on the third day from the operation, was very much inflamed, and the places of the sutures were disposed to ulcerate: a partial union of the integument had taken place, but it was to a very trifling extent. Her general appearance, upon the whole, was unfavourable: her pulse was quick and feeble, and the tongue dry and ulcerated. It was proposed to cut away the stitches, but, as the union was so very imperfect, it was apprehended that there would follow a protrusion of the small intestines; and so little sanguine were our expectations of a favourable result, that it was observed by Mr. Norman, there was very little fear of the stitches cutting their way through, for that they would last as long as the patient: at this time no other expectation could be reasonably entertained. The inflammation of the wound, which was of the erysipelatous kind, continued to increase, and in the course of eight or ten days had produced a most extensive sloughing, —a complete destruction of skin, cellular membrane, and fascia, for several inches around the wound. The colon was exposed for the whole extent of the incision into it; its edges were everted, its colour was a deep-red, and it was found to have contracted a firm adhesion to the muscular margin by which it was surrounded. During this sloughing process, the wound was poulticed, and the patient took bark, ammonia, and aromatic confection, port-wine, beef-tea, and purgatives; the mouth, tongue, and fauces, which were covered with aphthæ and ulcerations, were frequently washed with a solution of alum in honey and water, by means of sponge fastened to a piece of whalebone.

The inflammation, which had nearly covered the abdomen and loins, had abated, and the sloughing had terminated in about a fortnight after the operation. There was then a large, ill-conditioned, open wound; and the sloughing of the cellular membrane and fascia under the skin was so extensive that a probe would scarcely reach the termination of it in any direction. The powers of the constitution were so reduced, that there was very little hope of the healing of a wound so extensive: it did, however, heal slowly; and the union of the surfaces of a large pouch, which extended from the wound up to the ribs and towards the back, was promoted by a seton.

During the first four or five weeks after the operation, the patient was unable to void her urine voluntarily, which was therefore drawn off with a catheter, commonly twice a-day. She had, in the course of the healing of the wound, rigors, which were followed by fever, tenderness of the abdomen, sometimes vomiting; and she suffered several attacks of erysipelatous inflammation, which sometimes nearly covered the back, loins, nates, and posterior parts of the thighs. Her back became excoriated, and very troublesome from the irritability of the skin, from lying constantly upon it, and from the impossibility of keeping her always dry, as feces were almost constantly discharged.

With great labour and assiduity, the case, notwithstanding every possible impediment and disadvantage which could oppose recovery, slowly improved in its circumstances. When her general health was good, the wound healed rapidly; but there was seldom a steady amendment for many days together: fever, erysipelatous inflammation, sickness, or something, occurred to render recovery protracted. It was a ticklish and precarious business, and required throughout a very nice steerage. She was fortunate in having an excellent nurse, by whose constant and indefatigable care in cleaning the wound, removing dirty clothes, &c. great additional mischief was prevented. The feces appeared to be very irritating to the skin: it is probable that much of the inflammation might be imputed to this quality.

If any additional proof were required of the completeness of the obstruction, and of the necessity of the operation in this case, it may be found in the circumstance that the patient had no evacuation whatever from the natural anus during the first three months after the commencement of the obstruction. As she was constantly under the influence of aperient medicines, the contents of the bowels were always fluid, and their peristaltic movements preternaturally excited: it may therefore be inferred, as the continuity of the intestine was preserved, that, unless the obstruction had been in a degree to render the gut during this time totally impervious, some portion of the fluid contents of the bowels would in so long a period have descended into the rectum, from whence it would have been expelled; as clysters had been, which, to the amount of four or five ounces, were seldom retained longer than half an hour. My conjecture on the circumstance of there being for so long a time no evacuation from the natural anus, was, that the gut was impervious at the place of the obstruction, and that the portion of intestine below the wound had contracted, from defect of distention, and would in time become obliterated; the more readily, as its internal surface is naturally disposed for an active absorption. This conjecture was, however, erroneous, as conjectures are apt to be. On the 1st of October, more than three months from the commencement of the obstruction, an indurated and elongated portion of feces passed from the natural anus: the same thing has since occurred irregularly. These evacuations are, however, so scanty and extenuated, that there is at present but little reason to hope that the natural calibre of the gut will ever be restored.

The artificial opening has not shown lately any disposition to contract. The edges of the incision of the gut, in the course of the first fortnight, had united for a very trifling extent: the opening, therefore, towards the lower part is intersected by a point of union, leaving a large aperture above, and a small one below: the feces are, of course, principally discharged through the former. About a month since, she had an attack of apoplexy, which was followed by complete paralysis of the right arm. She was bled, purged, and blistered; the arm was frequently immersed in hot water, and rubbed with a stimulating liniment: she recovered the use of her arm in about four days, and her health was again as good as formerly. A fortnight after this attack, she complained of pain, vertigo, sense of weight in the head, and had had a restless night: ten ounces of blood were taken from the back of the neck by cupping, followed by a dose of calomel, salts, and senna. In a few days she was again free from complaint.

The artificial anus has now been established between five and six months: the object of the operation has been completely attained. The patient's general health is apparently good, and she has had no return of the dysenteric symptoms before mentioned. Her pulse is commonly about 70; her tongue clean, her complexion florid; her digestion is good, and so well disposed to make the most of her food, that her plan of diet is still directed to be one of rather strict abstinence. She has recovered her flesh in a great degree. She is able to sit up, or walk about the house: the latter, however, is an experiment which she has not ventured often. She has generally one or two stools a-day; and she does not, upon the whole, experience so much inconvenience from the manner of the evacuation as might have been expected. The bowel, when she is upright, has a great disposition to project: this would not have been the case if the skin covering it had not unluckily sloughed. The projection of the bowel does not amount to a considerable prolapsus: it is merely a distention of the portion which is exposed. It is expected that this disposition to prolapsus will be restrained when use has made her contrivances a little more perfect. She has a truss, somewhat similar to that for exomphalos, constructed with a circular spring, and a large pad containing a weak spiral spring, which is preserved in its place by means of straps: this contrivance has not, however, yet answered so well as a compress, confined by a band, pinned tightly around her.

At the time of making this artificial anus, it was projected rather upon the analogy afforded by that which sometimes succeeds to a strangulated hernia, than from any certain knowledge of precedents. I have since found that an incision was first made into the colon by M. Durêt, a surgeon at Brest, in a child born with an imperforate anus, in whom the termination of the rectum could not be discovered by an incision in the natural situation of this opening. The report of this case descends to the twenty-fifth month, when the child was suffering from inversion (*double renversement*) of the gut, which it was not possible, as it is stated, either to prevent or correct. This case, which is, I believe, the first and only one on record, is related by Sabatier.* Mr. Callisen, surgeon of Copenhagen, has proposed to discover the left side of the colon in its course in the lumbar region, where he supposes it to be on the outside of the peritoneum, and to make an incision into it between the edge of the false ribs and the crista of the ilium, parallel to the anterior edge of the quadratus lumborum muscle. This operation was performed by Mr. Callisen on the dead subject with difficulty; an example which is not likely to be followed on the living one, as it does not promise any particular advantage. I had, a few weeks since, the pleasure of seeing Mr. Freer (to whom surgery was already indebted for the first successful operation of tying the external iliac artery) in Bath; and he then confirmed the fact, which I had heard reported vaguely by some and contradicted by others, of his having made an incision into the colon with a view to the formation of an artificial anus. Mr. Freer has been so obliging as to favour

---

* *Médicine Opératoire*, tome troisième, p. 336.

me with the report of his experience in this operation, which want of leisure, want of health, or want of inclination, had hitherto prevented his making public himself: and, as he has added to the obligation by allowing me to make any use of his cases which I may think proper, giving the detail in his own words, I am happy in the opportunity of comprising in this paper the whole of our experience on an operation which is likely to prove an useful addition to the resources of surgery.

"On the 24th of December, 1817, I was requested to meet in consultation Dr. Johnstone and Dr. De Lys, of Birmingham, and Mr. Short, surgeon of Solihull, in the case of Mr. Lowe, a respectable farmer, residing about seven miles from Birmingham. Mr. Lowe was about forty-seven years of age, remarkably temperate and regular in all his habits, and had enjoyed uninterrupted good health till about fifteen months ago, when he began to suffer from dyspepsia, attended with a deficient secretion of bile. Dr. Johnstone had prescribed for him occasionally, with temporary advantage, mercurial medicines, so as to produce slight affection of the gums, and he had made use of gentle tonics: he had also taken the Leamington water. At the time I saw him, he complained of a fixed dull pain at the lower part of the belly, and was a good deal troubled with flatulence and other dyspeptic symptoms; he was exceedingly costive, and his feces were compressed in a very unnatural manner; he had also a slight difficulty in voiding urine.

"On introducing the finger *per anum*, we ascertained that there existed a contraction of the rectum, but situated so high that it could scarcely be reached by the end of the finger. The prostate gland was considerably enlarged. It was agreed in consultation, that frequent clysters should be administered, and that mechanical means should be resorted to, to overcome, if possible, the stricture of the rectum. Accordingly, rectum-bougies were employed regularly for several weeks. The patient seemed to improve in his general health, and was able to use moderate exercise, in attending to his business. Still he had great difficulty in voiding his stools, and their appearance showed that the rectum was as contracted as ever. Mr. Short informed us that he could not pass the bougie above five inches up the gut; and the disease was evidently gaining ground. Under these circumstances, Dr. De Lys and myself were sent for on the 30th of January, when we met Mr. Short, who gave us the above account of the failure of the means hitherto tried. The bougie could not pass beyond the contraction, and clysters had ceased to produce their accustomed effect.

"It was plain that the disease had made progress: the patient, having had no stool since the 27th, was exceedingly uneasy, and his bowels were very tense, though not in more pain than usual.

Small doses of elaterium were prescribed, and stimulating clysters of aloes directed; but without success.

"On the 3d of February, we were again sent for. The patient had passed no stool, and had vomited, which was very unusual with him. His belly was swelled and hard, but not tender to the touch; his pulse quick, and his countenance expressive of considerable anxiety. We again examined the rectum, but without being able fairly to reach the stricture; and no bougie, not even of the smallest size, could be made to pass through the contracted part of the gut. Under these circumstances it was thought right that an attempt should be made, by means of a bistoury, to relieve the stricture of the gut. I introduced a long bistoury,* guided by the fore-finger, along the hollow of the sacrum, in the best manner I could, till I met with a resistance which prevented the further progress of the instrument. All my attempts, however, to divide the stricture of the gut proved ineffectual; and, after passing a little wind and some blood, but no feculent matter, the patient was put into bed again; had an opiate ordered him, and a warm-bath. On the following day, we found him tolerably free from pain; he had passed a better night than we could have expected, and was free from sickness: still he had had no stool, although he had taken several doses of castor-oil, and had had emollient clysters administered to him.

"It now became evident that no relief was to be expected from the use of medicine. Death, in his present state, was inevitable; and we therefore determined to propose to him, as a last resource, an operation, which certainly was hazardous, but which his desperate condition appeared to justify. This operation consisted in making an incision through the parietes of the abdomen, in the left iliac region, so as to expose the colon near its sigmoid flexure, and to make an opening in that intestine for the purpose of establishing an artificial anus in that situation. We did not know, with any degree of certainty, what might be the nature of the obstruction of the rectum, for it was situated beyond the reach of the finger; but it was possible that the obstruction might be caused by the pressure of a tumor or of an abscess, or by some other cause not of a permanent nature, which might be removed in the course of time; and, in that case, the feces would return to their natural channel, and the artificial anus would close. But, even though so desirable an event should not happen, and though Mr. Lowe might have to submit, for the remainder of his days, to the

* The instrument I employed can scarcely be called a bistoury, being rather a flat trocar about six inches long, which was introduced within the canula, till it met the obstruction; the blade was then projected by means of a spring, and used as a bistoury.

loathsomeness and inconvenience of an artificial anus, still his life, which was most valuable to his numerous family, was worth preserving on any terms; and he expressed his willingness to submit to any operation which might give him a chance of life.

"The possibility of such an operation had occurred to Dr. De Lys and myself two years before, in attending a child born without an anus, and in whom it was found impossible, by means of a trocar, to reach the rectum. It struck us, in this case, that we should be justified in forming an artificial anus, in the manner I have described, as the only means of saving the life of the child. I accordingly performed the operation: a considerable quantity of meconium was evacuated; and, during the three weeks that the child lived, the feces passed freely at the wound. The child sucked and slept well, and seemed free from suffering, but died apparently from marasmus, the wounded gut adhering firmly to the wound of the parietes of the abdomen, and without any appearance of inflammation of the bowels or other viscera. In this case, though ultimately unsuccessful, the operation had undoubtedly prolonged the life of the child, and seemed to justify us in recommending it in the present instance. We knew, from the various cases recorded by surgical writers, that wounds of the colon are, *cæteris paribus*, less dangerous than those of any other intestine; and therefore we apprehended little or no danger from the actual injury done to the colon by the operation, and so far considered the colon to be the fittest situation for an artificial anus. Besides, it was evident that an artificial anus in the colon, near its sigmoid flexure, would interfere less with the functions of nutrition than if it were formed in any part of the small intestines.

"This operation we thought it right to recommend as a last resource; but, as the symptoms were not yet very urgent, it was deemed prudent to delay it till the increase of danger from the continuance of the obstruction should, if possible, still more fully justify us in proposing an operation so formidable, and of which the success must evidently be very doubtful. We did not choose, therefore, to recommend the operation that evening; but gave the patient to understand that, in case every other resource failed, we should, when we next saw him, think it our duty to propose his submitting to an operation.

"On the following day, February the 5th, we received a letter from Mr. Short, stating that Mr. Lowe had passed a very restless night, had vomited several times, was very much troubled with the hiccup, and complained of considerable soreness all over the belly; and was, he added, very anxious for our arrival, having made up his mind to submit to any operation that might be deemed advisable. We had, for some days past, looked forward to the probability of having to perform this operation, and had therefore, as much as possible, considered every circumstance connected with it, and had agreed that the most eligible spot for making the external incision that was to expose the colon, would be the left iliac region, about an inch above the anterior superior spine of the ilium, and between one and two inches in front of that process. I accordingly made an incision nearly three inches in length in this situation; and, after cautiously dividing the abdominal muscles and the peritoneum, I exposed the colon covered by the omentum: the omentum was with some difficulty pushed aside by the finger, as it felt adhering to the intestines; and, after confining the colon with one stitch at each end of the wound, I made a longitudinal incision into it, about two inches in length. There was an immediate gush of a considerable quantity of liquid and highly fetid excrementitious matter, and a great deal of wind escaped from the wounded intestine; the belly became soft, and, though Mr. Lowe complained of uneasiness from the wound, he said the pain was considerably diminished. He was ordered to be put into a warm-bath in the course of a few hours, and Mr. Short was requested to stay with him all night.

"On introducing the fore-finger into the cavity of the abdomen before opening the gut, we ascertained that the bowel thus exposed was of considerable size, so as to give us reason to presume that it was the colon, of which we could distinctly feel the longitudinal band. The outward incision had been purposely made as small as possible, from a fear that the bowels, being excessively distended, would burst through the wound with great violence. We had been so much troubled by this accident, in operating on the intestines of dogs, that we dreaded it exceedingly; but Mr. Lowe bore the operation so calmly, that no such accident occurred, and we were enabled to make the most deliberate examination of the gut, before proceeding to lay it open. After the wounded intestine had been plentifully evacuated, spontaneously in the first instance, and then by an injection of warm water, I introduced my fore-finger up and down the gut, and the direction of its course upwards and backwards towards the left kidney, and downwards towards the sigmoid flexure of the colon, confirmed me in the opinion that I had succeeded in exposing the colon.

"On the 6th of February, the day after the operation, Mr. Lowe had, since we left him the day before, passed through the wound several dark-coloured liquid stools, and a good deal of wind. The belly was soft and free from soreness; but he complained of excessive pain in his loins; his pulse was quick, and his tongue furred. An aperient mixture of rhubarb and magnesia was prescribed, and he was directed to take an opiate at bed-time.

"On the 7th, Mr. Short informed us that Mr. Lowe had complained of considerable soreness of the belly; and that, his pulse having become sharp, he had been induced to take about twelve ounces of blood from his arm. The pain had been relieved by the bleeding, and the pulse, though quick, was become soft. He still complained of the most intolerable pain in the loins; he voided urine, however, very freely. The wound looked well, and there had been a free discharge of thin feculent matter from the artificial anus. There was a constant rumbling of wind in the belly, which however felt very soft, and was not sore to the touch.

"On the 8th, as the stools had not been voided so freely since yesterday, and as the belly, though not sore, was rather tense, it was thought right to administer a clyster of warm water through the wound of the colon: the liquid passed very freely and without pain, but brought away no feculent matter. The pain in the loins continued unabated: he was troubled with an almost incessant hiccup, and with considerable thirst; the tongue was dry and furred, and the pulse quick. A purge of calomel and rhubarb was prescribed, and an opiate at night; and he was directed to take occasionally, whenever the hiccup was particularly distressing, a table-spoonful of a medicine consisting of the camphorated mixture, with paregoric elixir and sulphuric ether.

"On the 9th.—The laxative had operated freely: he had taken an opiate at bed-time, but had passed a very restless night. The hiccup continued most distressing.

"On the 10th.—No material alteration had taken place in any respect. The gut adhering very evidently to the wound, I thought it right to remove the ligatures, which were become loose. He still continued very restless at night.

"The 11th.—Mr. Lowe had passed a rather better night, but was exceedingly dejected, with a look of considerable anxiety in his countenance. Hitherto he had had no appetite, nor could he be said now to have a natural one; but he complained of a sense of sinking at his stomach, which was relieved only by taking food very frequently, in small quantities. His diet, for a long time, and especially since the obstruction had become so inveterate, had been liquid and of the lightest and most nutritious quality, and his beverage of the mildest kind, such as whey, toast and water, or imperial. Since yesterday he had, at his own request, feeling exceedingly languid, been allowed to take a little wine and water. His pulse was very quick and weak; his tongue clean, but dry, with intolerable thirst and clamminess of the mouth.

"The 12th.—Mr. Lowe appeared better, and had passed a more quiet night. He had voided several stools, some similar in appearance to meconium, and others light-coloured and greenish, like the stools of an infant. The hiccup continued very troublesome. He had voided, *per anum*, a great quantity of a very dark-coloured fluid, which had the appearance of blood in a putrescent state, mixed with a small quantity of thin excrementitious matter. He was ordered to take an aperient mixture with tincture of rhubarb.

"The 13th.—He had passed a very disturbed night, and, though he had had a little sleep, he did not feel refreshed by it, and thought he had not slept at all. He appeared more anxious and dejected than ever, and had lost all hope of recovery. His pulse was very quick and feeble, and his skin cold and clammy. He was excessively thirsty, had no appetite, but still continued to take small quantities of brandy and water or wine and water. He had voided several stools similar to those mentioned in yesterday's report. The state of Mr. Lowe was now such as to leave no hope of his recovery; still, as the belly was rather swollen, and the stools very unnatural, it was thought right to repeat the tincture of rhubarb, so as to keep up the action of the bowels, and promote the free expulsion of wind and feces, which always gave him relief. The gut since yesterday had become everted, like a prolapsed anus, about three inches in length; but was easily replaced, and supported by a soft compress with gentle pressure.

"The 14th.—As Dr. De Lys and myself were proceeding to Solihull, we were met by a messenger from Mr. Short, who informed us that Mr. Lowe was in a dying state. We sent to request that Mr. Short would take the earliest opportunity after the decease of Mr. Lowe, to apply for permission to examine his body; but neither Mr. Short's solicitation, nor our personal application to Mr. Lowe's relations, could obtain for us the permission we so anxiously wished for."

Thus, it appears that, of four instances, the formation of an artificial anus, by making an opening into the colon, has been completely successful in two: the one in a case of imperforate anus, and the other in a case of insurmountable obstruction from disease; and, in the other two, the degree of success was so considerable, that their unfavourable termination will perhaps be regarded as a probable exception than anticipated as a general result. Reverting to the case of my patient, Mrs. White, it may be worth while to observe, that the inconveniences of an anus in this situation are not such as to have caused her any regret for having submitted to the operation: on the contrary, so far from her having any reason to lament this circumstance, I believe myself that it has afforded her a moral, as well as a physical, advantage; for she is now at no loss for an interest, and is provided with something to think of for the rest of her life.

*Bath; December 12th,* 1820.

